# Hypercalcemia episodes caused by ectopic parathyroid adenoma and subsequent gastrointestinal stromal tumor: A case report and literature review

**DOI:** 10.3389/fonc.2025.1720028

**Published:** 2025-12-17

**Authors:** Yi-Ching Lin, Jen-Chieh Lee, Chen-Yu Wen, Wei-Yih Chiu

**Affiliations:** 1 Department of Internal Medicine, National Taiwan University Hospital and National Taiwan University College of Medicine, Taipei, Taiwan; 2 Department of Pathology, Graduate Institute of Pathology, National Taiwan University Hospital, National Taiwan University College of Medicine, Taipei, Taiwan; 3 Department of Internal Medicine, National Taiwan University Hospital, Taipei, Taiwan; 4 Department of Laboratory Medicine, National Taiwan University Hospital, Taipei, Taiwan

**Keywords:** ectopic parathyroid adenoma, gastrointestinal stromal tumor, hypercalcemia, imatinib, primary hyperparathyroidism, tyrosine kinase inhibitor

## Abstract

**Objective:**

Ectopic parathyroid gland-induced hypercalcemia is unusual, whereas hypercalcemia from a gastrointestinal stromal tumor (GIST) is extremely rare. This study aims to present a rare case of simultaneous ectopic parathyroid adenoma and GIST, associated with two episodes of hypercalcemia, and to review imaging techniques for ectopic parathyroid localization and the mechanism of hypercalcemia linked to GISTs.

**Methods:**

The clinical manifestations, diagnostic workup, therapeutic interventions, and outcomes of the present case were analyzed. To evaluate advanced imaging modalities, particularly four-dimensional computed tomography (4D-CT) and ^18^F-fluorocholine (FCH) PET/CT, for ectopic parathyroid localization, a PubMed search for literature in English from inception to July 2025 was conducted using the terms (“4D-CT” AND “ectopic parathyroid”) or (“^18^F-fluorocholine PET/CT” AND “ectopic parathyroid”). Additional keywords related to parathyroid imaging, including “FCH-PET/CT”, “^18^F-fluorocholine PET/CT”, and “4D-CT”, were incorporated to broaden the search. Reports of GIST-related hypercalcemia were also identified to summarize underlying mechanisms and management approaches.

**Results:**

An 87-year-old man presented with progressive renal dysfunction and hypercalcemic hyperparathyroidism. ^99m^Tc-sestamibi single photon emission computed tomography/computed tomography (SPECT/CT) identified an ectopic parathyroid lesion in the anterior mediastinum, which was successfully treated with video-assisted thoracoscopic surgery, resolving hypercalcemia. Two years later, recurrent hypercalcemia occurred with reduced parathyroid hormone levels. A CT scan and biopsy revealed a GIST in the pelvis, an extremely rare cause of hypercalcemia. Imatinib normalized calcium and parathyroid hormone levels and induced tumor regression. Nineteen reports showed that 4D-CT or FCH-PET/CT successfully localized ectopic parathyroid lesions after conventional imaging modalities were inconclusive. In 9 cases of GIST-associated hypercalcemia, pathophysiology may involve parathyroid hormone–related protein (PTHrP) or 1-alpha-hydroxylase, with glucocorticoids having a potential role in treatment.

**Conclusions:**

To our knowledge, this case represents the first reported coexistence of an ectopic parathyroid adenoma and a GIST. 4D-CT and FCH-PET/CT can be used as alternative imaging modalities following ^99m^Tc-sestamibi SPECT/CT to locate ectopic parathyroid lesions. The mechanism behind GIST-related hypercalcemia may involve the expression of PTHrP or 1-alpha-hydroxylase in tumor tissues.

## Introduction

Primary hyperparathyroidism (pHPT) is a common endocrine disorder, with estimated incidences of 233 and 85 per 100,000 women and men in the United States, respectively (1). Ectopic parathyroid glands are present in 10% to 22% of pHPT cases (2, 3), likely due to abnormal embryonic migration. Accurate preoperative localization of ectopic parathyroid adenomas is crucial for optimizing surgical outcomes, and various imaging modalities are available to aid in this process (4). As for gastrointestinal stromal tumors (GISTs), they have an annual global incidence of approximately 1.5 per 100,000, affecting both sexes equally with a peak incidence at age 70 (5). GISTs, the most common type of soft tissue sarcoma, arise from the digestive tract and are primarily driven by gain-of-function mutations in the *KIT* or *PDGFRA* genes, which encode receptor tyrosine kinases. The introduction of tyrosine kinase inhibitors has significantly improved the prognosis of this previously chemotherapy-resistant cancer (6).

To date, there are few case reports on hypercalcemia associated with GISTs (7–15). The coexistence of an ectopic parathyroid adenoma and a GIST with two separate episodes of hypercalcemia in a single patient is exceptionally rare. This study aims to present this rare condition and to review the advanced imaging techniques used for localization of ectopic parathyroid lesions and the pathophysiology of GIST-induced hypercalcemia.

## Case presentation

**Figure 1:**
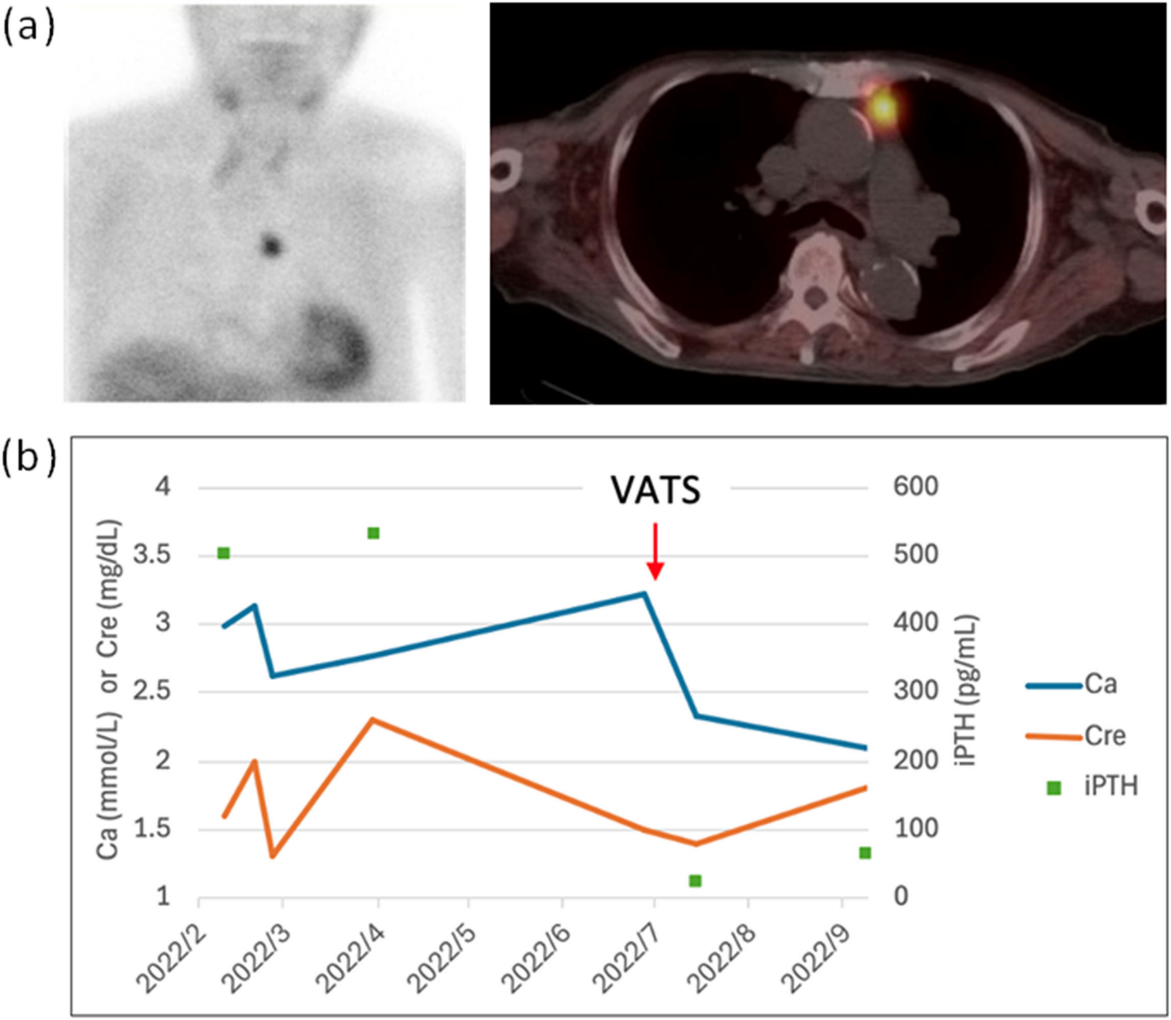
First episode hypercalcemia (PTH-dependent). ^99m^Tc-sestamibi SPECT/CT identified an ectopic parathyroid adenoma in the anterior mediastinum **(a)**. The lesion was successfully removed via video-assisted thoracoscopic surgery, leading to resolution of the first, PTH-dependent, episode of hypercalcemia **(b)**. Only serum calcium and creatinine were tested preoperatively; PTH was not included.

An 87-year-old man with a medical history of heart failure with preserved ejection fraction, chronic kidney disease, hypertension, and cerebellar infarction presented with worsening renal function and hypercalcemia in 2022. His regular medications included furosemide 40 mg daily and trichlormethiazide 2 mg daily. There was no family history of endocrine disorders or tumors. The serum calcium level at the time was elevated at 2.99 mmol/L (normal range: 2.15-2.58 mmol/L). Laboratory evaluation showed a serum phosphorus level of 3.0 mg/dL (normal range: 2.5-5.0 mg/dL), markedly elevated intact parathyroid hormone (PTH) level of 505.1 pg/mL (reference range: 15.0-68.3 pg/mL), a 24-hour urine calcium level of 304.8 mg/day, and a high calcium clearance ratio of 0.06, leading to a diagnosis of primary hyperparathyroidism. Bone mineral density showed osteopenia, and renal sonography revealed a small left renal stone. A neck ultrasound did not identify any parathyroid lesion. A ^99m^Tc-sestamibi single photon emission computed tomography/computed tomography (SPECT/CT) revealed a tracer-avid lesion in the left superior anterior mediastinum, which was successfully excised via video-assisted thoracoscopic surgery (VATS), resulting in normalization of serum calcium during follow-up (**Figure 1**). The excised lesion measured 3.0 × 2.6 × 1.2 cm and weighed 5.1 g. Histologic examination revealed an ectopic parathyroid gland with hyperplasia, and immunohistochemistry was positive for PTH in the lesional cells.

**Figure 2:**
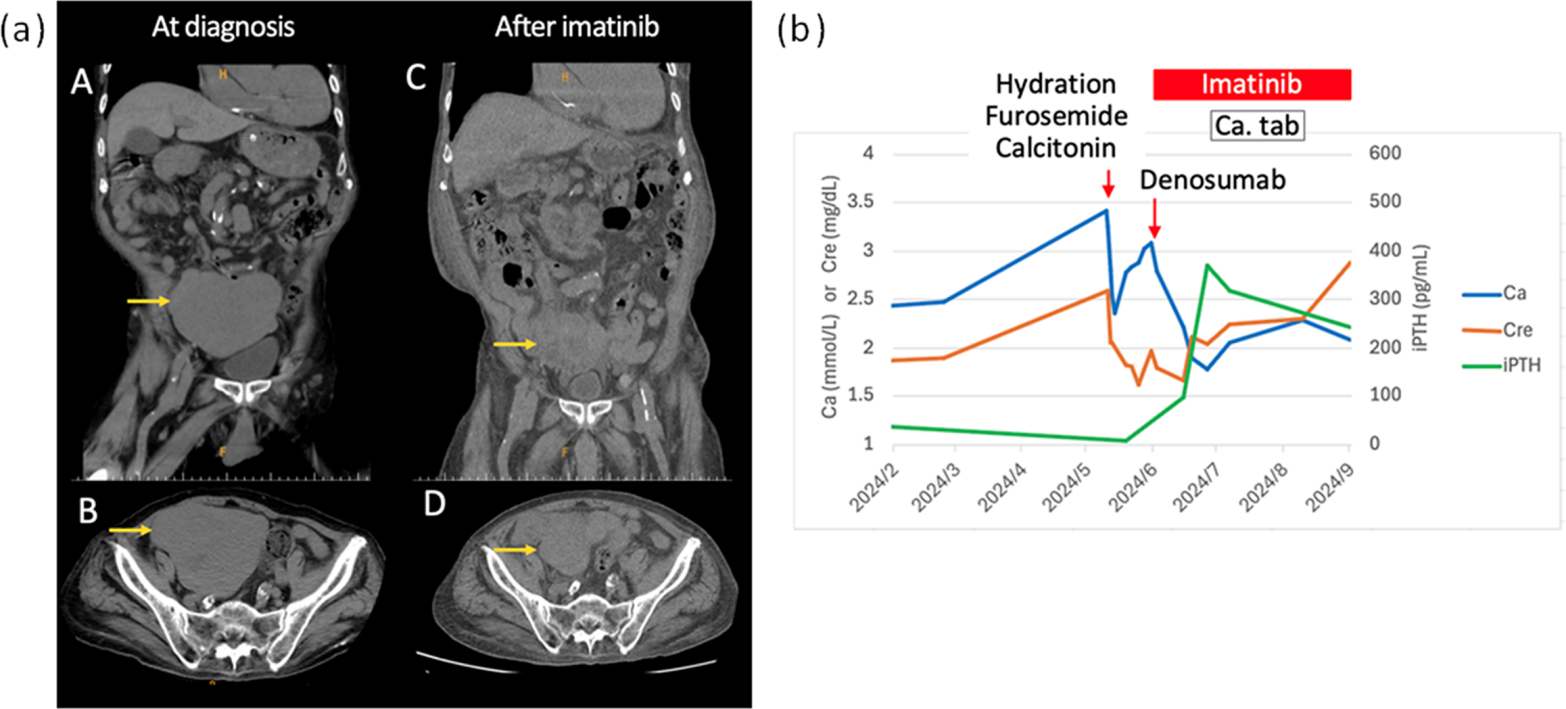
Second episode hypercalcemia (PTH-independent). The initial pelvic mass measured 12.8 cm at diagnosis. After three months of imatinib treatment, the tumor size decreased to 8.8 cm **(a)**. During the second, PTH-independent, episode of hypercalcemia, calcium levels normalized within two weeks of imatinib therapy for the gastrointestinal stromal tumor. Transient iPTH elevation due to hypocalcemia resolved with calcium supplementation **(b)**.

**Figure 3:**
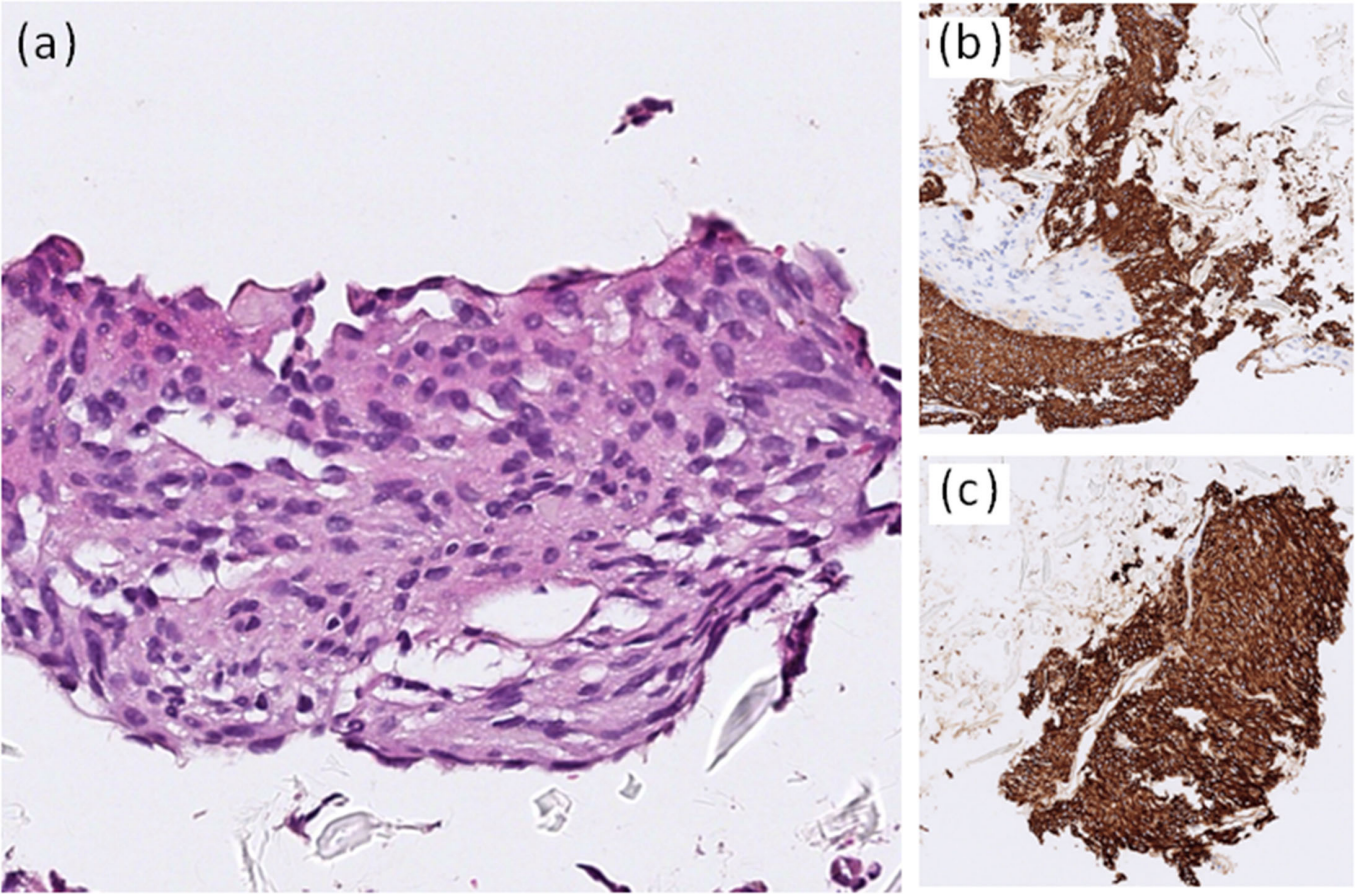
Histopathologic findings of the pelvic gastrointestinal stromal tumor. A spindle cell neoplasm shows spindle-shaped tumor cells with monomorphic nuclei and eosinophilic cytoplasm (30×) **(a)**. Tumor cells show diffuse positive staining for C-kit/CD117 (12×) **(b)** and DOG1 (12×) **(c)**.

Two years after the prior episode of hypercalcemia, he presented with generalized weakness, drowsy consciousness, and abdominal distension, prompting his transfer to our emergency department. Physical examination revealed muscle strength graded 4/5 in all extremities. Laboratory tests showed hypercalcemia (3.31 mmol/L; normal range: 2.15-2.58 mmol/L), acute kidney injury (creatinine: 2.91 mg/dL; normal range: 0.6-1.3 mg/dL), and a suppressed intact PTH level of 9.3 pg/mL (reference range: 15.0-68.3 pg/mL), with normal phosphorus, magnesium, and liver function levels. The serum 25-hydroxyvitamin D was slightly low at 17.3 ng/mL. His home medications included furosemide 40 mg once daily for heart failure. A non-contrast computed tomography (CT) scan of the abdomen and pelvis revealed a 12.8 cm soft tissue mass in the pelvic cavity (**Figure 2a**). The lesion was further characterized by a MRI with contrast showing intermediate-to-low T1WI signal, heterogeneous high T2WI signal, diffusion restriction, and heterogeneous contrast enhancement. These findings are consistent with a GIST. Whole body bone scintigraphy showed no skeletal metastases. A ultrasound-guided biopsy was performed. Histologically, the specimen shows a spindle cell neoplasm with nor pleomorphism or necrosis, and mitoses are absent (<1/50 HPF). Immunohistochemistry demonstrates diffuse positivity for CD117 and DOG1 and negativity for SMA, desmin, and S100, confirming the diagnosis of a GIST, with a Ki-67 index of 10% (**Figure 3**). Based on tumor size, low mitotic activity, and uncertain origin, the lesion meets a high-risk category by NIH consensus criteria. Although AFIP (Miettinen–Lasota) assessment is limited, the large size and low mitotic rate suggest intermediate-to-high risk. The tumor corresponds to cT4N0M0, stage IIIA. Given the suppressed intact PTH, elevated serum calcium, and a large GIST, malignancy-associated hypercalcemia was strongly suspected.

The patient was initially treated with intravenous fluids, furosemide, and calcitonin. Hypercalcemia was incompletely resolved, with a corrected calcium level of 3.02 mmol/L. A single dose of subcutaneous denosumab (120 mg) and imatinib therapy (100 mg daily) were administered on the same day following pathological confirmation of GIST (**Figure 2b**). The imatinib dose was adjusted due to the patient’s advanced age, acute decompensated heart failure, and an estimated creatinine clearance of 20 mL/min. Calcium levels normalized two weeks after denosumab and imatinib administration. Over the next four weeks, the intact PTH level, which had transiently increased due to hypocalcemia, returned to normal with calcium supplementation (**Figure 2b**). The effect of imatinib on calcium normalization was confounded by denosumab, which can cause hypocalcemia and transient PTH changes. After three months of imatinib therapy, normocalcemia was maintained without calcium supplementation and tumor size was reduced on a follow-up CT scan (**Figure 2a**), supporting the diagnosis of GIST-associated hypercalcemia. The dosage of imatinib remained unchanged during therapy, and no clinically significant adverse effects were observed.

## Discussion

To our knowledge, this is the first reported case demonstrating the coexistence of an ectopic parathyroid adenoma and a GIST associated with two separate episodes of hypercalcemia. In animal studies, certain genetic mutations, such as *mafB*, have been linked to the development of ectopic parathyroid tissue (16). In contrast, GISTs are associated with genetic alterations in *KIT* and *PDGFRA* (6). Currently, there is no evidence to suggest that these two conditions arise from shared genetic mutations. Although no known gene directly links parathyroid neoplasms and GISTs, both conditions predominantly affect individuals over the age of 50 and can independently cause hypercalcemia, which may explain their co-occurrence in this case.

Hypercalcemia with an elevated or inappropriately elevated PTH level is indicative of PTH-dependent hypercalcemia. A thorough medical history and physical examination, including an assessment of symptoms like kidney stones, bone pain, or abdominal issues, are essential to identify underlying causes. Additional tests may include a calcium/creatinine clearance ratio and a 24-hour urine calcium excretion to assess for familial hypocalciuric hypercalcemia, a serum creatinine test to evaluate renal function, and a measurement of 25-hydroxyvitamin D levels to assess for vitamin D deficiency. For the localization of hyperfunctioning glands in pHPT, imaging techniques have traditionally involved neck ultrasound and ^99m^Tc-sestamibi SPECT/CT (1). Other imaging modalities, such as four-dimensional computed tomography (4D-CT) of the neck, magnetic resonance imaging (MRI), positron emission tomography/computed tomography (PET/CT) with radiotracers such as ^18^F-fluorocholine (FCH), and parathyroid venous sampling (PVS) are typically reserved for cases with negative or inconclusive results from conventional methods (17–19). A network meta-analysis (20) of 8,495 patients from 119 studies demonstrated that FCH-PET/CT is superior to the CT category and conventional modalities like ultrasound and MIBI after 2010; however, the selection of preoperative imaging may vary based on availability, cost, and performance.

Ectopic parathyroid adenoma should be suspected in pHPT patients who exhibit an ectopic lesion on imaging studies, negative localization of the parathyroid gland, or persistent disease after surgery (1). Ectopic parathyroid glands have been classically described as occurring anywhere from the angle of the mandible to the mediastinum. They most commonly occur in several locations, including the retro/paraesophageal space, mediastinum, intrathymic or intrathyroidal sites, carotid sheath, and a high undescended cervical position (17). Their wide range of locations can make them challenging to identify accurately. Neck ultrasound is particularly effective for locating intra-thyroid parathyroid glands, but is less accurate for detecting ectopic glands in the mediastinum (3). In contrast, ^99m^Tc-sestamibi SPECT/CT are particularly effective in identifying ectopic glands, especially in the thymus, mediastinum, or retroesophageal space, with a reported sensitivity of 89% (3). Despite the growing adoption of contemporary tools like 4D-CT and FCH-PET/CT in parathyroid imaging, their effectiveness in individuals with ectopic parathyroid glands remains poorly understood. To date, FCH-PET/CT has successfully localized ectopic parathyroid adenomas in six reported cases, five of which had failed prior localization with ^99m^Tc-sestamibi SPECT/CT (21–25) (**Table 1**). Similarly, 4D-CT enabled detection in fifteen cases after conventional imaging modalities, including ultrasound, ^99m^Tc-sestamibi SPECT/CT, CT, MRI, and PVS, were inconclusive (**Table 1**), highlighting their critical role in challenging cases (26–39). By contrast, it is impossible to determine whether cases successfully localized by conventional imaging modalities were actually missed by advanced imaging tools like 4D-CT or FCH-PET/CT. Further research is needed to determine the optimal imaging modality for the localization of ectopic parathyroid adenomas.

**Table 1:** Successful 4D-CT and FCH-PET/CT localization of ectopic parathyroid glands.

Author/country/year	Sex/age	Clinical features	Ca	iPTH	Adenoma size	Failed imaging	Successful imaging	Location	Pathology
FCH-PET/CT									
Triantafyllidou/Switzerland/2018 (21)	F/40, Pregnant	Nausea, hypotension	3.04 mmol/L	120 pg/mL	6 mm	US	MIBI, FCH	anterior mediastinum	intrathymic parathyroid adenoma
	M/60	Weakness, weight loss, paraesthesia, anorexia	3.6 mmol/L	349 pg/mL	N/A	US, MIBI	FCH	right superior mediastinum	adenoma
Hai/China/2022 (22)	F/52	Bone pain	Free Ca 3.38 mmol/L	734.89 pg/mL	2×1×1 cm	US, MIBI	FCH	left thyroid lobe	intrathyroidal parathyroid adenomatous hyperplasia
Jang/Korea/2022 (23)	F/22	Hypercalcemia in medical check-up	11.4 mg/dL	256.5 pg/mL	1.8 cm	US, MIBI, MET	PVS, FCH	retropharyngeal space	adenoma
Tawil/Colombia/2023 (24)	F/36	Bone pain	iCa 1.65 mmol/L	497 pg/mL	2.5 cm	US, MIBI, Neck& chest CT	FCH	submandibular region	adenoma
Aphale/India/2024 (25)	F/32	Fatigue, backache, gastritis, palpations	10 mg/dL	3039 pg/dL	7×7×20 mm	US, MIBI	FCH	submandibular region	adenoma
4D-CT									
Welling/USA/2011 (26)	F/47	Persistent pHPT, fatigue, memory loss, muscle pain	N/A	N/A	right 600 mg, left 360 mg	US, MIBI	4D-CT	bilateral retropharyngeal space	hyperplasia
Dhillon/USA/2013 (27)	M/12.5	Fatigue, muscle pain	11.4 mg/dL	130 pg/mL	280 mg	US, MIBI	PVS, 4D-CT	anterior mediastinum	adenoma
Elhelf/USA/2017 (28)	M/58	Fatigue, knee pain, renal stones	10.9 mg/dL	99.7 pg/mL	1.32 g	US	MIBI, 4D-CT	middle mediastinum	adenoma
Kim/USA/2017 (29)	F/68	Renal stones	10.8 mg/dL	112.8 pg/mL	N/A	US, MIBI	4D-CT	right pyriform sinus	adenoma
	F/58	Osteoporosis, renal stone, fatigue, memory loss	12.4 mg/dL	109 pg/mL	5×4 mm	US, MRI, MIBI	4D-CT	right pyriform sinus	adenoma
Binnetoğlu/Turkey/2020 (30)	F/51	Asymptomatic hypercalcemia	12.9 mg/dL	63.9 pg/mL	1.5 ×1 cm	US, MIBI	4D-CT	anterior mediastinum	adenoma
Hsieh/USA/2020 (31)							MIBI, 4D-CT	right piriform sinus	adenoma
Unais/India/2020 (32)	M/55	Hyperparathyroidism	N/A	2400 pg/mL	2 cm	US, MIBI	4D-CT	behind the left common carotid artery	adenoma
Flokas/USA/2020 (33)	F/11	Asymptomatic hypercalcemia	14.1mg/dL	230 pg/mL	21×16×11 mm		MIBI, 4D-CT	anterior mediastinum	intrathymic parathyroid adenoma
Badhe/India/2023 (34)	M/13	Hip pain	11 mg/dL	2081 pg/mL	2.1×1.6 cm	US	MIBI, 4D-CT	anterior mediastinum	adenoma
Mwewa/Belgium/2023 (35)	N/A/72	Recurrent pHPT post-thyroidectomy	2.56 mmol/L	174 ng/L	11 mm	^99m^Tc-tetrofosmine	MET, 4D-CT	anterior mediastinum	adenoma
Zenno/USA/2023 (36)	F/9	Nausea, vomiting, constipation	12.1 mg/dL	70 pg/mL	7×5 mm	MIBI	4D-CT	piriform sinus	adenoma
Edamadaka/India/2024 (37)	F/53	N/A	N/A	N/A	N/A	US	MIBI, 4D-CT	submandibular area	adenoma
Naik/India/2025 (38)	M/20	Acute pancreatitis	11.5 mg/dL	1559 pg/mL	24×12×19 mm	US	neck MRI, 4D-CT	anterior mediastinum	adenoma
Sandakly/Lebanon/2025 (39)	M/60	Renal stones, constipation	12.8 mg/dL	240 pg/mL	7×7×13 mm	US, MIBI	chest CT, 4D-CT	aortopulmonary window	adenoma

4D-CT, four-dimensional computed tomography of the neck; FCH, ^18^F-fluorocholine PET/CT; M, male; MET, ^11^C-Methionine PET/CT; MIBI, ^99m^Tc-sestamibi SPECT/CT; N/A, not available; PVS, parathyroid venous sampling; pHPT, primary hyperparathyroidism; US, ultrasound.

Hypercalcemia with a reduced PTH level is suggestive of PTH-independent hyperparathyroidism. For PTH-independent hypercalcemia, further evaluation typically involves imaging studies such as chest radiograph, mammogram, or CT scans to rule out malignancy, bone scans to assess for bone metastases or primary bone tumors, and serum and urine protein electrophoresis to evaluate for multiple myeloma. Additional tests may include serum parathyroid hormone–related protein (PTHrP) and serum 1,25-dihydroxyvitamin D levels to assess for malignancy or vitamin D toxicity. The diagnostic flow is then tailored based on the results, with further evaluation and testing as needed to identify the underlying causes of hypercalcemia.

Hypercalcemia induced by GISTs is an extremely rare condition, with only 9 reported cases to date (7–15), as demonstrated in **Table 2**. Reported cases in GIST-induced hypercalcemia involved patients aged 45 to 77 years, with a female-to-male ratio of six to three. Almost all previously reported patients presented with advanced-stage disease and initially received treatment with tyrosine kinase inhibitors rather than surgery (7–13, 15). In GIST-induced hypercalcemia, earlier studies suggested that PTHrP secretion by the tumor was the primary mechanism (8, 12). However, recent literature increasingly implicates a 1,25-dihydroxyvitamin D–mediated pathway (10, 11, 13, 15). GISTs have been shown to produce 1-alpha-hydroxylase (15), which converts 25-hydroxyvitamin D into its active form, 1,25-dihydroxyvitamin D. This excess active vitamin D subsequently increases intestinal calcium absorption and bone resorption, leading to hypercalcemia. In patients presenting with hypercalcemia, standard hypercalcemia management, along with targeted cancer treatment for GISTs, has been employed. Additionally, three case reports have explored the use of prednisolone for acute hypercalcemia management, with doses of 1.5 mg/kg and 37.5 mg per day, respectively. Calcium levels successfully decreased in two cases (11, 15), while one case showed no response (13). We were unable to definitively elucidate the mechanism of hypercalcemia in our case due to the unavailability of PTHrP/CYP27B1 (1-alpha-hydroxylase) immunohistochemical staining and serum assays for PTHrP and 1,25-dihydroxyvitamin D at our institution. Further research is necessary to determine the underlying mechanisms, the efficacy of steroid therapy, and appropriate dosing strategies.

**Table 2:** The clinical manifestation and possible mechanism of documented cases with hypercalcemia induced by GISTs.

Author/country/year	Sex/age	Clinical features	Primary site	Metastatic sites	Bone metastasis	Ca	iPTH	1,25-(OH)_2_D	PTHrP
Al-Moundhri/Oman/2006 (7)	M/45	Abdominal pain, anorexia, weight loss, vomiting	Pelvic mass	Peritoneum, liver	N/A	3.02 mmol/L	—	—	—
Beckers/Netherlands/2007 (8)	F/48	Abdominal pain, weight loss, anorexia, night sweats	Abdominal mass, exact site uncertain	Peritoneum, liver, omentum	N/A	3.28 mmol/L (2.10-2.55)	<1.0 pmol/L (<6.8)	—	66 pmol/L (<2.5)
George/Malaysia/2008 (9)	F/65	Confusion	Probably small bowel	Liver, abdominal wall	N/A	4.1 mmol/L (2.10-2.60)	—	—	—
Jasti/USA/2013 (10)	F/52	Confusion, hallucinations, weight loss, abdominal pain	Pelvic mass	Peritoneum, liver, lung	Negative	14.3 mg/dL (8.5-10.5)	1 pg/mL (10-65)	^$^83 pg/mL (18-78)	<2.4 pmol/L (0-4)
Hygum/Denmark/2015 (11)	F/70	Weight loss, fatigue, palpable abdominal mass	Duodenum	Liver	Negative	(iCa) 2.11 mmol/L (1.18-1.32)	1.5 pmol/L (1.6-6.9)	375 pmol/L (60-180)	<1.9 pmol/L (<2.6)
Urasaki/Japan/2016 (12)	M/73	Not recorded	Small bowel	Peritoneum, possible gastric metastasis	N/A	hyper-	—	—	38.9 pmol/L (<1.1)
Barbaryan/USA/2017 (13)	F/77	Weakness, confusion	Abdominal mass, exact site uncertain	Peritoneum, liver	Negative	15 mg/dL (8.5-10.6)	7.9 pg/mL (10-65)	129 pg/mL (19.9-79.3)	normal
Hart/UK/2018 (14)	M/50	Generalized abdominal pain, anorexia, weight loss	Abdominal mass, exact site uncertain	No metastasis	Negative	3.67 mmol/L	1.1 pmol/L	—	—
Herrera-Martinez/Spain/2022 (15)	F/66	Abdominal pain, weight loss, anorexia	Abdominal mass, exact site uncertain	No metastasis	Negative	15.4 mg/dL	12.8 pg/mL (14-72)	119 pg/mL (18-71)	<1.1 pmol/L

^$^laboratory data obtained one month after initiation of imatinib.

Data in parentheses are reference values provided in original papers.

1,25-(OH)_2_D, 1,25-dihydroxyvitamin D; iPTH, intact parathyroid hormone; N/A, not available; PTHrP, parathyroid hormone-related protein.

## Conclusions

This is the first reported case of concurrent ectopic parathyroid adenoma and GIST-induced hypercalcemia. In this case, ^99m^Tc-sestamibi SPECT/CT successfully localized the ectopic parathyroid adenoma. In diagnostically challenging cases where conventional imaging modalities are inconclusive, 4D-CT and FCH-PET/CT may serve as complementary tools for localization of ectopic parathyroid adenomas. GIST-related hypercalcemia is rare and may result from tumor secretion of PTHrP or 1-alpha-hydroxylase–mediated overproduction of 1,25-dihydroxyvitamin D, often presenting with suppressed PTH levels. Management includes standard calcium-lowering agents and GIST-directed therapies. Steroids may help in calcitriol-mediated cases, but their efficacy remains uncertain.

## Data Availability

The original contributions presented in the study are included in the article/supplementary material. Further inquiries can be directed to the corresponding author.
